# Factors associated with the confirmation and death for Brazilian spotted fever in an important endemic area of the State of São Paulo, 2007-2021

**DOI:** 10.1590/0037-8682-0617-2023

**Published:** 2024-07-29

**Authors:** Jardel Brasil, Rodrigo Nogueira Angerami, Maria Rita Donalisio

**Affiliations:** 1 Secretaria de Saúde de Americana, Unidade de Vigilância em Saúde, Americana, SP, Brasil.; 2 Universidade Estadual de Campinas, Programa de Pós-Graduação em Saúde Coletiva, Campinas, SP, Brasil.

**Keywords:** Brazilian spotted fever, Lethality, Predictive factors, Diagnosis

## Abstract

**Background:**

We evaluated the predictive factors for case confirmation and death from Brazilian spotted fever in an endemic area of Southeastern Brazil.

**Methods:**

A cross-sectional study was conducted. All suspected cases reported between 2007 and 2021 were analyzed using two logistic regression models.

**Results:**

60 cases were confirmed. Male sex, age group of 40-59 years, tick parasitism, presence of capybaras or horses, exanthema and hospitalization were positively associated with confirmation. Death was associated with a longer period between first symptom-hospitalization and shorter treatment.

**Conclusions:**

Complete clinical evaluation and information on risk exposure are key to early suspicion, diagnosis, treatment and prevention of deaths.

Brazilian spotted fever (BSF) is an endemic rickettsiosis in the state of São Paulo and is associated with high case-fatality rates in the region (average, 54.9%)^1^ and compulsory nationwide notification^2^. BSF is transmitted by *Amblyomma* ticks and caused by the gram-negative bacterium *Rickettsia rickettsii.* In the initial stages, BSF presents with non-specific symptoms (fever, headache, and myalgia), which makes early diagnosis difficult, and can progress to severe forms with potential for high lethality^3^.

The capybara (*Hydrochoerus hydrochaeris)* is one of the main hosts of *Amblyomma sculptum, the main BSF vector,* and plays the role of *Rickettsia* amplifiers in the epidemiology of BSF in several endemic areas in Brazil^4^. 

According to the current epidemiological classification criteria^5^, the municipality of Americana (inner São Paulo State, Brazil) and neighboring areas, present sites classified as transmission areas (identified as probable sites of infection -PSIs), risk areas (with presence of vector ticks, significant presence of sentinel animals seroreactive for *Rickettsia*, and human frequency), and alert areas (with presence of vector and human frequency, but significant absence of seroreactive animals). This region is at risk of human infection with high lethality, presence of hosts and vectors. Therefore, the investigation of ecoepidemiological characteristics of transmission areas and factors associated with the severity of cases can contribute to combating BSF as a public health problem. This study aimed to analyze the epidemiological profile and evaluate potential predictive factors for BSF case confirmation and progression to death in a relevant endemic area. 

This cross-sectional analytical study evaluated the main clinical and epidemiological variables associated with laboratory confirmation and predictive factors for death in BSF cases in the Americana region between 2007 and 2021. The Americana municipality (244,370 inhabitants) is located in the eastern region of the state of São Paulo in Southeastern Brazil. The city has a Human Development Index of 0.81 and is crossed by the Piracicaba River Basin (Piracicaba, Capivari, and Jundiaí rivers), PCJ Basin^6^.

Data of all suspected BSF cases (residents and non-residents) notified by the municipal health department or with PSI confirmed in Americana during the study period were analyzed. The Health Surveillance Unit of Americana provided access to the database of the National Notifiable Diseases Information System and Epidemiological Investigation Reports.

A suspected case of BSF was defined as an individual with sudden onset fever, headache, myalgia, a history of tick bite and/or contact with domestic and/or wild animals, and/or having attended a known area of transmission of BSF during the past 15 days or patients with sudden onset fever, headache, and myalgia, followed by maculopapular exanthema, between the second and fifth days of evolution and/or hemorrhagic manifestations^2^.

All reported suspected cases (confirmed and discarded), residents and non-residents, were included, not only the autochthonous ones, since the PSI of cities conurbated with Americana belong to the same river basin and share similar ecoepidemiological transmission scenarios. The cases were confirmed by laboratory criteria according to the recommendations of the Brazilian Ministry of Health through indirect immunofluorescence antibody assay, PCR, immunohistochemistry, and/or isolation of *Rickettsia* in cell culture insulation, all performed at a public health reference laboratory^2^. 

The following variables were studied: demographic (age, sex, and race/color), clinical (signs and symptoms), epidemiological (frequency in risk area, presence of host animals and tick parasitism, exposure at home, leisure, or work environment), clinical management/treatment (occurrence of hospitalization and dates of hospitalization and discharge), laboratory (date of serum sample collection and technique used for diagnosis), and case conclusion (final classification, confirmation/discard criteria, autochthonous/imported case and evolution to healing or death, and date of death).

For categorical variables, absolute and relative frequencies were calculated and chi-square (χ^2^) or Fisher's exact association tests (or its generalization) were performed. Continuous variables were analyzed using mean comparisons, standard deviations, and t-tests after checking the normality assumptions. All the tests were performed at a statistical significance level of 5%.

After bivariate analysis, two logistic regression models were adjusted, with the outcomes “confirmed case of BSF” (Yes/No) and “death” (Yes/No), including covariates with p < 0.20 in the bivariate analysis. Regression models were adjusted via stepwise method, estimating odds ratios and confidence intervals (95%CI). The independent variables that presented p ≤ 0.05 at the end of the analysis remained in the models.

Statistical analyses were performed using *IBM SPSS Statistics for Windows, Version 25.0 (IBM Corp., Armonk, NY, EUA).* This study was approved by the Ethics in Research Committee of the School of Medical Sciences of the University of Campinas (Process no. 5.474.734).

From 2007 to 2021, 507 suspected cases of BSF 397 (78.3%) residents and 110 (21.7%) non-residents were reported by the epidemiological surveillance system of Americana. Among all suspected BSF cases, 60 (11.8%) were confirmed (35 [58,3%] were autochthonous 25 [41,7%] were non-autochthonous) and 447 (88.2%) were discarded. The non-autochthonous cases were from neighborhood municipalities that share areas bathed by the PCJ Basin, with the presence of dams, lagoons, rivers, and streams, a previously described epidemiological scenario^7^. These results reinforce the importance of classifying areas not only under the perspective of autochthonous transmission municipalities, but also based on ecoepidemiological risk scenarios that are often common to neighboring municipalities, even those without confirmed cases.

We found a higher frequency among men (91.7%), of working age (73.6%), and age group of 40-59 years (43,3%), as reported in other endemic areas for BSF^7^ ^,^ ^8^. However the involvement of children aged 0-9 years with 12 individuals (20%) stands out **(** Table 1 **)**. This fact can be potentially explained by the frequency of children and adolescents in transmission areas during leisure activities in the company of family members. 

**TABLE 1:** Demographic, clinical, and risk exposure variables of suspected BSF cases associated with diagnostic confirmation through multiple logistic regression observed in Americana, São Paulo between 2007 and 2021.

	Confirmed		Discarded		Total		OR _adjusted_ (95%CI)*
	N	%	N	%	N	%	
**Sex**							3.35 (1.12-10.02)
male	55	91.7	322	72.0	377	74.4	
female	5	8.3	125	28.0	130	25.6	
Age**							1.03 (1.01-1.04)
0 to 9 years	8	13.3	98	21.9	106	21.0	
10 to 19 years	4	6.7	73	16.3	77	15.2	
20 to 39 years	12	20.0	131	29.3	143	28.2	
40 to 59 years	26	43.3	101	22.6	127	25.0	
60 years or more	10	16.7	44	9.9	54	10.6	
**Risk exposure**							
parasitism by tick	38	63.3	236	52.8			2.53 (1.21-5.31)
Presence of animals in PSI***							
capybara	37	61.7	57	12.8			5.31 (2.41-11.63)
horse	28	44.7	43	9.6			5.59 (2.31-13.59)
**Clinic**							
exanthema	21	35.0	73	16.3	94	18.5	4.96 (2.19-11.27)
hospitalization	53	88.3	189	42.3	242	47.7	11.6 (4.55-29.57)

*OR: Odds Ratio; **95%CI:** 95% Confidence Interval. **Reference category age 0-9 years. ***Probable Site of Infection.

Another variable associated with case confirmation was the presence of capybaras and horses in the PSI, as observed in other endemic areas^7^, a condition that evidences the risk of transmission, an important information for the diagnostic suspicion. A risk scenario was established; that is, the ecoepidemiological context of environments altered by anthropogenic activities, with the presence of degraded riparian forests, dirty pastures, and high population densities of capybaras, which is a mammal amplifier of *Rickettsia* ^9^. These animals, along with horses, are the main hosts of *A. sculptum.* However, horses do not develop rickettsemia that allows the infection of new ticks and does not act as an amplifier of *Rickettsia,* but they are considered efficient sentinel animals for detecting the circulation of bacteria because of the humoral response presented to parasitism by infected ticks^10^.

The urbanization of BSF, characterized by the expansion of transmission in peri-urban and urban areas, puts a higher proportion of the population close to the disease transmission cycle. The presence of urban populations of capybaras and high environmental infestation by vector ticks increase the risk of parasitism by infected ticks in humans and potential transmission in peridomiciliary, workplace, and nearby leisure areas, including public parks^11^ ^,^ ^12^. These scenarios highlight changes in the ecoepidemiology of BSF transmission patterns from rural areas to peri-urban and urban areas and a potential increase in number of cases.

The presence of *A. sculptum* and *A. dubitatum* in the riparian areas of Americana has been registered in systematic acarological research with the objective of assessing the level of environmental infestation of ticks and their infection by *Rickettsia* ^*12*^. In these investigations, both species presented similar annual distributions for the immature stages, which are the main stages involved in the transmission of *R. rickettsii* in the Metropolitan Region of Campinas^3^. The fact that larvae and nymphs parasitize humans in areas of BSF transmission by *A. sculptum* may partly explain the absence of parasitism reported in 38.3% of the confirmed cases. Due to their small size, these immature forms of the vector tick are difficult to visualize and perceive by affected individuals. Therefore, a significant proportion of patients may not have a documented history of parasitism, which makes clinical suspicion difficult.

The maculopapular exanthema, characteristic of the infection, was predictive of confirmation, helping in the differential diagnosis of BSF. Hospitalization, conjunctival hyperemia, and shock are clinical conditions associated with advanced stages of confirmed disease severity (Figure 1). The high hospitalization (88.3%) and case fatality (58.3% overall; 65.7% considering only autochthonous cases) rates suggest potentially late clinical suspicion and/or or an inappropriate time to begin the recommended treatment. The lack of early detection of cases in the first days of the disease may compromise the start of immediate treatment, even when suspected, as recommended, leading to the worsening of clinical signs and, consequently, contributing to higher hospitalization rates and mortality^13^ ^,^ ^14^.

**FIGURE 1:**
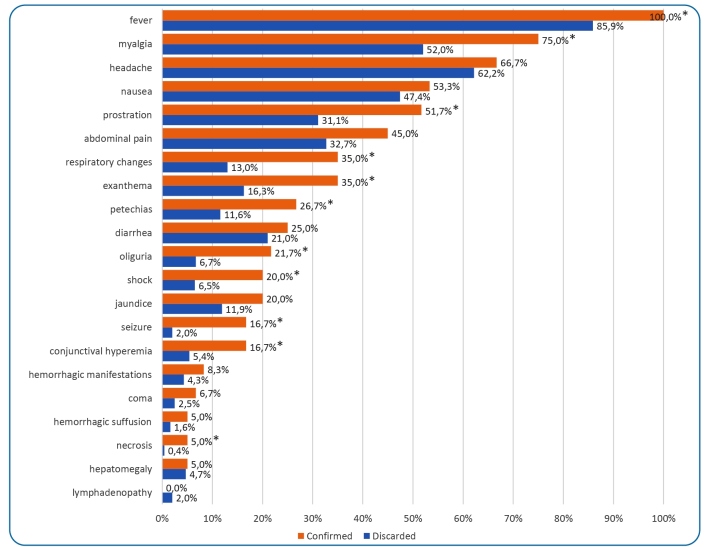
Main clinical signs presented by suspected cases of Brazilian spotted fever reported in Americana, São Paulo between 2007 and 2021. *Clinical signs that presented statistical difference between confirmed and discarded cases in bivariate analysis.

The case fatality rate for the period was above the state average, which is already considered high^1^. This situation may be related to late diagnostic suspicion, also evidenced by the short interval between the onset of treatment and fatal outcomes (Table 2), which often occurs only after the third medical care^8^. At this point, the clinical signs have already worsened and the opportunity to start early treatment with specific antibiotic therapy was lost^14^. Additionally, the high lethality observed in Americana may be largely due to an outbreak that occurred in this municipality in 2018^8^. BSF outbreaks have been associated with high case fatality rates above the expected average. The high percentage of diagnostic confirmation using the PCR test (58.3%) suggests a delay in etiological confirmation, occurring after death, as this test is usually performed by the reference laboratory in fatal cases.

**TABLE 2:** Time elapsed (mean number of days and standard deviation) from the date of onset of symptoms until hospitalization, collection of exams, and overall hospitalization and treatment time for confirmed cases of Brazilian spotted fever in Americana, São Paulo between 2007 and 2021.

Time period	Total	Healing	Death	p*
First symptoms to hospital admission	4.3±3.5	3.5±3.4	4.9±3.4	0.048
First symptoms to first sample collection for IFA**	5.7±3.6	5.7±3.7	5.7±3.5	0.799
Overall hospitalization time	7.7±6.4	9.6±9.0	6.7±4.2	0.308
Overall treatment time	3.2±5.4	8.3±7.5	1.2±2.4	<0.001

Obs: *p value for t-test; **Indirect Immunofluorescence Antibody assay.

Some limitations of this study include the use of secondary data with missing information, such as clinical outcomes, including those related to specific treatments. In addition, the absence of complete information makes it difficult to analyze more comprehensive sociodemographic variables, such as schooling, profession, and race/color, reported by affected individuals. 

Important predictive factors for the confirmation of cases of spotted fever were evidenced, suggesting the need for greater awareness among health services for the occurrence of the disease in the region. The diagnostic suspicion based only in the clinical features is a challenge due to the nonspecific symptoms of the initial clinical signals, similar to other high circulation febrile diseases, such as arboviruses, leptospirosis, among others. Therefore, in addition to a complete clinical evaluation, healthcare professionals must be aware regarding epidemiological scenario and risk exposures to vector parasitism and infection^3^ ^,^ ^15^. Early clinical suspicion and timely treatment can potentially limit the worsening of the disease, need for hospitalization, and risk of death. Qualified clinical evaluation and assessment of exposure to the risk of tick parasitism are essential to reduce severe cases, sequelae, and the high lethality currently observed among cases.

In this context, public health agents in endemic areas need to know and monitor the clinical and epidemiological characteristics of BSF for contributing to improve the assistance provided to suspected cases and consequently reduce the high fatality rates associated with the disease. Furthermore, understanding of the risk of the disease must be reinforced through educational prevention actions among patients and their family members. Improving knowledge about epidemiological surveillance and the clinical pattern of BSF by healthcare professionals in endemic areas represents a fundamental strategy to reduce both lethality, based on early suspicion and correct treatment, and the risk of infection, through disease prevention and control actions.
